# It is Unlikely That Influenza Viruses Will Cause a Pandemic Again Like What Happened in 1918 and 1919

**DOI:** 10.3389/fpubh.2014.00039

**Published:** 2014-05-07

**Authors:** Liting Song

**Affiliations:** ^1^Hope Biomedical Research, Toronto, ON, Canada

**Keywords:** general health, highly pathogenic virus, host immunity, influenza virus, pandemic, socio-economic factor

Influenza and influenza viruses are well-known popular topics to medical professionals and the general public. Influenza viruses had caused a pandemic globally during 1918 and 1919, and that influenza pandemic had taken away more than 20 million people’s lives in the world. However, in my opinion, it is unlikely that influenza viruses will again cause a pandemic on a level (both of the morbidity rate and the mortality rate) comparable to what happened in 1918 and 1919.

Influenza viruses very easily reassort, recombine, and point mutate in nature due to their segmented RNA genome structures, however, unlike highly pathogenic (virulent) viruses like rabies virus, Lassa fever virus, smallpox virus, eastern equine encephalitis virus, Ebola virus, Marburg virus, and human immunodeficiency virus 1 (HIV-1); most influenza viruses (wild types and mutants) are moderately pathogenic. The case fatality rates of some highly virulent viruses and related references are listed in Table 1.

**Table 1:** **Case fatality rate of some highly pathogenic viruses**.

Virus	Case fatality rate	Reference
Rabies virus	Almost 100%	(1)
Lassa fever virus	5–10%	(2)
Smallpox virus (variola major)	<10–100%	(3)
Eastern equine encephalitis virus	36%	(4)
Ebola virus	50–89%	(5)
Marburg virus	83%	(6)
Human immunodeficiency virus 1	Almost 100% except a few persons who carry a gene mutation	(7)

On November 11, 1918, the fighting of World War I was stopped, and World War I was officially ended on June 28, 1919 with the signing of the Versailles Treaty. It is estimated that around 8.5–10 million soldiers lost their lives in World War I due to battle. The war also directly caused more than 6 million civilian deaths. Millions of people suffered from hunger and malnutrition during the war. Malnutrition weakened the human immune system and made a person more vulnerable to infectious diseases like tuberculosis and influenza, therefore, hunger and malnutrition were indirectly responsible for millions of deaths in the world in that period of time. For example, about 700,000 Germans died from malnutrition-related diseases in the years of 1914–1918. During the 1918–1919 influenza pandemic, between 21 and 25 million people died of influenza worldwide. Those people were killed both directly and indirectly by influenza virus infections. Many families were too poor to buy food and coal, and to afford health care expenses when their family members were ill. Influenza virus could infect all members of a family, and this could result in no one left to feed the fires, and to prepare food for the whole family, even if they had firewood, coal, and food left in their homes. Sadly, a large number of people died of influenza virus infections along with starvation, cold, and poor living conditions (8).

In recent years, while hunger and malnutrition are not major and serious problems in some developed countries anymore, they are still very difficult to overcome in many developing countries. In these less-developed countries, there were approximately 925 million people who suffered from hunger; 125 million children were underweight; and 195 million children were stunted each year (9). Nevertheless, in comparison to 1918 and 1919, currently, we have much better social and economic conditions and public health systems globally; and generally speaking, the majority of people in the world have better nutritional and educational statuses; better living and working conditions; therefore, better general health and immunity. Furthermore, in 1918 and 1919, physicians and nurses almost had nothing in their hands to help individuals who were infected by influenza viruses. Today, although we still do not have very effective, powerful, and practical anti-influenza drugs available, we at least have some improved, useful, and helpful anti-viral drugs like zanamivir, and effective, convenient anti-cold medicines like Tylenol or Advil. We do not have a universal vaccine to prevent all influenza virus infections, but we can make effective vaccines to a specific influenza virus strain in a short time. Actually, in the United States of America, the influenza classed mortality rate declined from 10.2/100,000 in the 1940s to 0.56/100,000 in the 1990s; and the classed mortality rates of 1957–1958 and 1968–1969 influenza pandemics were not remarkably different from the non-pandemic seasons (10).

Because of the above reasons, we can optimistically assume that even the same strain of influenza virus, which caused pandemic in 1918 and 1919, would not be able to kill millions of people and cause a pandemic comparable to the 1918–1919 pandemic again in the future.

Additionally, a significant number of viruses can cause influenza-like syndromes, such as rhinovirus, parainfluenza virus, adenovirus, coronavirus, respiratory syncytial virus, Coxsackie B virus, echovirus, and metapneumovirus (11, 12). Some of the above-mentioned viruses like adenovirus and mutated coronavirus could cause problems that are comparable to influenza viruses (13, 14).

The World Health Organization (WHO) mistakenly raised the level of influenza pandemic alert from phase 5 to the highest phase 6 on June 11, 2009 (15). However, the truth was that most cases of H1N1 influenza A virus infections were mild, the symptomatic case fatality rate was only 0.005% in New Zealand (16); and in New York City, the case fatality rate was 0.0094–0.0147% for persons ≥65 years old, and for those of 0–17 years old, the case fatality rate was 0.0008–0.0012% (17). Some researchers argued that it should not have been called an influenza pandemic in the first place if the clinical severity was considered (15, 18–20). I believe it was unwise that we had paid too much attention to influenza viruses for the past many years. Moreover, people would be tired of the alerts made by the WHO like what had happened in the story of “The Boy Who Cried Wolf” (21), and when a real wolf – an epidemic caused by a highly pathogenic virus like Lassa fever virus, Ebola virus, Marburg virus is occurring, people in the world might not believe WHO’s new alerts anymore, and this would cause serious problems.

Nature has taken its course, the bird influenza epidemic in China was limited and controlled successfully, because there were only 140 isolated laboratory confirmed cases of patients in a long period of time of 2013 (March 29, 2013–December 5, 2013), and H7N9 influenza A virus had not spread to other countries (two patients were infected in mainland China and were diagnosed in Taiwan and Hong Kong later) (22, 23). Not surprisingly, every year there would be some influenza patients and a few of them would die from the infections, as it is almost impossible to eliminate influenza viruses from the natural environment in many years. The severity of a viral infection is determined by both of the viral virulence (pathogenicity) and the host immunity. Some researchers’ opinions on H7N9 avian influenza virus were incorrect and/or inadequate. They mainly focused on influenza viruses and worried about viral mutations, viral pathogenicity, viral adaptation, and transmission. They overestimated the negative part of socio-economic factors of the present east China: overcrowded population in the epidemic region; very busy national and international transportation and travel; a large number of live poultry markets … but they underestimated the currently changed, developed, and improved positive part of socio-economic factors in China. The following factors might be used to explain why that H7N9 influenza A virus epidemic was limited and controlled in China, and only a few immunocompromised patients were killed by H7N9 influenza A virus. First, China has a relatively organized and effective public health system, there are four levels of (national, provincial, prefectural-level city, and county) centers for disease control and prevention all over China (24). Second, physicians and nurses in China were prepared and knowledgeable of influenza virus infections. Third, samples from patients with suspected influenza virus infections were collected and sent to the local and national centers for disease control and prevention promptly. H7N9 influenza A viruses were isolated and identified very quickly. Thereby, they were able to diagnose, confirm, and report three cases of H7N9 influenza patients in the early stage of the epidemic (24, 25). Fourth, health care and public health workers were protected properly. Consequently, none of the health professionals was infected by H7N9 influenza A virus in 2013. However, a surgeon died of H7N9 influenza in Shanghai, China in January of 2014 (26). Fifth, they detected H7N9 influenza A viruses from the samples of chickens, pigeons, and the environment of live poultry markets in Shanghai (27); and closed the live poultry markets of the involved epidemic region quickly. Sixth, patients were isolated and treated timely in hospitals, 74% (1251/1689) of those close contacts of H7N9 influenza patients were monitored and observed. Thus, H7N9 influenza A virus could not spread to a bigger population (24). Last but not least, we are connected to the Internet now, and it seems that our planet is much smaller today than the earlier days when we did not have the Internet, because communication and information exchange have become so fast, easy, and convenient presently. During that avian influenza epidemic, some influenza experts in the world shared/exchanged H7N9 influenza A virus information and provided professional consultations and suggestions efficiently and rapidly. All these public health routine practices and measures resulted in that H7N9 influenza epidemic being controlled and stopped in China (24). I have to point out that the cases of diagnosed H7N9 avian influenza A virus infection might only be the tip of the iceberg. Aside from one laboratory confirmed asymptotic case of H7N9 influenza A virus infection in Beijing (22), there were probably many undetected mild or asymptotic cases of influenza A H7N9 infection. The reason is that most people usually think a common cold is a very common and normal occurrence, and they don’t take flu-like illnesses seriously. In most situations, they would just stay home and take some medicines. Only those who have very severe flu-like symptoms would see doctors, and thereby be detected and diagnosed, accordingly the real case fatality rate should be much lower than the detected 32.14% (45/140, one case from Taiwan, and one case from Hong Kong) (22, 23).

Nowadays, we travel faster, and we travel more frequently and globally, and we have more complicated social activities and lifestyles, thereby increasing the chances of viral mutation; and we realize that influenza viruses are even easier to reassort, recombine, and mutate in nature than many other RNA viruses. However, we are now living in a technologically, economically, and socially much better and advanced society. I believe influenza virus infections are controllable and preventable, with the increased population health and immunity, with the WHO Global Influenza Surveillance and Response System, and with standard/routine epidemiological practices, and with new effective anti-viral agents and vaccines in production in the future. Now, I first predict that influenza viruses will unlikely again cause a pandemic on a level comparable to what happened in 1918 and 1919. Hopefully, one day we could consider a strategy to produce a universal vaccine that can prevent people from infections of all influenza virus strains, or we could produce some very effective anti-influenza virus drugs; then influenza would not be a problem anymore. We should learn lessons from the mistakes we made in the past. It is reasonable and necessary to be cautious about influenza viruses, but overreactions or catastrophic reactions should be avoided in the future. My opinion is anti-traditional; the purpose of this article is to influence public health policy, and to save some of the limited resources and money for more important diseases like heart diseases, cancer, diabetes, AIDS, hepatitises, and tuberculosis (15).

## Author Contributions

Liting Song: conception of manuscript, drafting of manuscript, critical revision of manuscript, and final approval of manuscript.

## Conflict of Interest Statement

The author declares that the research was conducted in the absence of any commercial or financial relationships that could be construed as a potential conflict of interest.
